# Concomitant Assessment of Oral and Gastric Microbiota Composition in Autoimmune Gastritis Patients: A Case–Control Study

**DOI:** 10.3390/microorganisms14040789

**Published:** 2026-03-31

**Authors:** Laura Belloni, Sophia Cingolani, Leonardo Mancabelli, Giulia Stendardo, Francesca Fabretti, Marica Vavallo, Giulia Pivetta, Emanuele Dilaghi, Gianluca Esposito, Bruno Annibale, Marco Ventura, Christian Milani, Edith Lahner

**Affiliations:** 1 Department of Medical-Surgical Sciences and Translational Medicine, Sant’Andrea Hospital, Sapienza University of Rome, 00185 Rome, Italy; laura.belloni@uniroma1.it (L.B.); sophia.cingolani@uniroma1.it (S.C.); giulia.stendardo@yahoo.com (G.S.); francesca.fabretti@uniroma1.it (F.F.); marica.vavallo@uniroma1.it (M.V.); giulia.pivetta@uniroma1.it (G.P.); emanuele.dilaghi@uniroma1.it (E.D.); gianluca.esposito@uniroma1.it (G.E.); bruno.annibale@uniroma1.it (B.A.); 2 Department of Chemistry, Life Sciences and Environmental Sustainability, University of Parma, 43124 Parma, Italy; leonardo.mancabelli@unipr.it (L.M.); marco.ventura@unipr.it (M.V.); christian.milani@unipr.it (C.M.)

**Keywords:** autoimmune gastritis, microbiota, gastric dysbiosis, hypochlorhydria

## Abstract

Autoimmune gastritis (AIG) in its advanced atrophic stage is characterized by reduced acid secretion, dysbiosis, and gastric cancer (GC) risk. Swallowed oral bacteria surviving in increased intragastric pH may play a carcinogenic role. Oral microbiota was linked to increased GC risk. In AIG, the concomitant assessment of oral and gastric microbiota has not yet been performed. This study aimed to investigate the oral and gastric microbiota in AIG patients to clarify the role of oral bacteria in gastric dysbiosis. A case–control study on *n* = 20 histologically diagnosed AIG patients and *n* = 20 controls without AIG is conducted. Saliva samples were obtained from subjects who were fasting and without toothbrushing. Within 1 h, gastroscopy with biopsies (for histopathology and DNA extraction) was performed. Saliva (*n* = 40) and biopsy (*n* = 40) samples were frozen at −20 °C. DNA was extracted and prepared; paired-end sequencing was performed (IlluminaMiSeq-sequencer, San Diego, CA, USA). Bacterial abundance in biopsies was higher in AIG than in controls (*p* = 0.06), but was not different in the saliva (*p* = 0.54) samples. In biopsies, AIG showed a lower Shannon-Index than controls (*p* = 0.001). In saliva studies, AIG showed a higher Shannon-Index than controls (*p* = 0.0). In biopsies, *Streptococcus oralis*, *Fusobacterium pseudoperiodonticum*, *Veillonella rogosae*, and *Gemella sanguinis* were more frequent in AIG (*p* < 0.03). The most abundantly shared taxa between saliva and biopsy were *S. oralis* and *Prevotella histicola*; *Gemella sanguinis*, *Fusobacterium pseudoperidonticum*, and *Veillonella rogosae* were shared in AIG patients only. This study confirmed gastric dysbiosis in AIG. Oral taxa were more commonly associated with AIG and shared between the mouth and the stomach. In AIG, the oral microbiota is associated with gastric dysbiosis, highlighting the importance of oral eubiosis in patients with impaired gastric acid secretion.

## 1. Introduction

Autoimmune gastritis (AIG) is a chronic, immune-mediated disorder characterized in its advanced stages by oxyntic mucosa atrophy. The immune system damages the oxyntic mucosa of the stomach through autoreactive T cells. This results in reduced secretion of hydrochloric acid, essential for iron and vitamin B_12_ absorption [1] and maintenance of the gastric acid barrier [2,3]. AIG is a preneoplastic condition that can potentially lead to gastric type I neuroendocrine tumors (gt1NET) and gastric cancer [4,5,6,7].

The oral and gastric microbiomes represent two distinct but interconnected microbial environments, both of which influence the local and systemic health of the host [8,9]. The oral bacteria may influence the gastrointestinal microbiota by translocating into the gastrointestinal tract with saliva and affecting the gastrointestinal homeostasis, and may be associated with inflammation and carcinogenesis [10]. The oral cavity is a major bacterial reservoir in the human body, hosting a consortium of microorganisms specifically adapted to inhabit the human mouth [11]. The oral microbiome has reported to play a role in dental and periodontal diseases [12,13]. A potential relationship between the oral microbiota and gastrointestinal diseases, in particular gastrointestinal cancers has emerged [14,15,16,17,18]. Moreover, a link between oral or periodontal disease and gastric cancer has been reported [19,20,21,22].

Also, the gastric microbiome has emerged as an important factor in the pathogenesis of various gastric diseases, including gastric cancer [8,13,23,24,25]. In physiological conditions, the highly acidic intragastric environment makes the colonization of the gastric mucosa challenging for general bacteria [2,3]. Historically, the stomach was considered sterile until the discovery of *H. pylori*, which could be isolated from the gastric mucosa [8,23]. In contrast, AIG leads to a substantial change in the intragastric microenvironment, i.e., a reduction in hydrochloric acid and an increased gastric pH, which fosters the overgrowth of swallowed oral or intragastric bacteria, potentially leading to gastric dysbiosis that may influence the host’s inflammatory response and play a crucial role in gastric pathogenesis. Gastric dysbiosis may play a role in the progression of gastritis to intestinal metaplasia and cancer [24,26,27,28].

To our best knowledge, studies concomitantly investigating the oral and gastric microbiota composition in patients with AIG are lacking. Therefore, the aim of the current study was to analyze the composition and potential differences and similarities of the oral and gastric microbiota in patients with AIG (cases) and without AIG (controls).

## 2. Materials and Methods

### 2.1. Study Design and Population

In this case–control study, we recruited consecutive subjects scheduled for gastroscopy in a dedicated gastrointestinal endoscopy session at an academic hospital for clinical suspicion of AIG due to anemia or dyspepsia or endoscopic surveillance of already diagnosed AIG (February–April 2023). From each subject, we collected before gastroscopy saliva samples (*n* = 40) and, during gastroscopy, biopsies for microbiota analyses (*n* = 40) and histopathological assessment of gastritis according to the updated Sydney system [29].

Inclusion criteria were: age more than 18 years, clinical suspicion of AIG due to anemia or uninvestigated dyspepsia or endoscopic surveillance in patients already diagnosed with AIG. Exclusion criteria were: age less than 18 years, incomplete gastroscopy, acute gastroenteritis or colitis less than two months before inclusion, treatment with proton pump inhibitors, antibiotics, probiotics or chemotherapy less than one month before inclusion, positivity to *H. pylori*.

Each patient filled in a clinical questionnaire including demographics, gender, nationality, region of residence, education level, employment status, lifestyle items (smoking, alcohol, physical activity, diet), body mass index, family history for gastric cancer, comorbidities, reason for gastroscopy, drugs, and items regarding oral hygiene (use of chewing gums or candies, daily tooth brushing, use of mouth-washing, last oral hygiene appointment, last dental check-up, removable or fixed dentures, history of dental or periodontal diseases). All patients enrolled in the study gave informed consent and the local ethical committee authorized the study (7295/2022).

### 2.2. Saliva Sample Collection

All included subjects were contacted by phone the day before the scheduled gastroscopy and informed about the instructions for saliva sample collection. Patients were instructed not to brush teeth, use mouthwash, or consume chewing gum/candies the morning of collection. Also, smoking was forbidden on the day of sample collection. After rinsing the mouth with water and waiting 30 min, participants provided a saliva sample in a sterile tube containing DNA-Shield (Zymo Research, Irvine, CA, USA), which stabilizes nucleic acids and preserves microbial integrity at room temperature. Samples were, however, immediately frozen at −20 °C.

### 2.3. Gastric Biopsies

During gastroscopy, one corpus mucosa biopsy (the first one taken) was reserved for microbiome analysis using sterile forceps and was immediately placed in a DNA-Shield-filled Eppendorf tube, which was immediately stored at −20 °C. A further five gastric biopsies were obtained according to the updated Sydney system: 2 from the antrum, 1 from the angularis incisura, and 2 from the corpus-fundus [30] and sent for histopathological assessment of gastritis.

### 2.4. Histopathological Assessment of Gastritis

Gastric biopsy samples were stained with hematoxylin and eosin (H&E) and examined by a pathologist with high expertise in gastric pathology. Each sample was evaluated for inflammation, atrophy, intestinal metaplasia, and *H. pylori* infection according to the updated Sydney system [29]. AIG diagnosis was based on corpus glandular atrophy with oxyntic gland loss with ECL cell hyperplasia and pseudopyloric and/or intestinal metaplasia, and with a spared antral mucosa. Anti-parietal cell autoantibodies were positive in all patients [1,30]. Controls were defined as subjects without AIG, having either a histologically normal gastric mucosa or antral-restricted, non-atrophic, non-active *H. pylori* negative gastritis (with a normal corpus oxyntic mucosa).

### 2.5. DNA Extraction and Sequencing

Each sample (saliva samples and gastric biopsies) was subjected to DNA extraction using the QIAmp DNA mini kit following the manufacturer’s instructions (Qiagen, Hilden, Germany). The extracted DNA was prepared using the Illumina Nextera XT DNA Library Preparation Kit and following the Illumina NexteraXT protocol. Specifically, DNA samples were enzymatically fragmented, barcoded, and purified using magnetic beads. Subsequently, samples were quantified using a fluorometric Qubit quantification system (Life Technologies, Carlsbad, CA, USA), then loaded on a 2200 Tape Station Instrument (Agilent Technologies, Santa Clara, CA, USA) and normalized to 4 nM. Paired-end sequencing was performed using an Illumina MiSeq sequencer with MiSeq Reagent Kit v3 (Illumina Inc., San Diego, CA, USA).

The resulting fastq files were subjected to filtering to remove low-quality and *Homo sapiens* reads using the METAnnotatorX2 software, version 1.0, following the standard filtering parameters reported in the manual (DOI:10.1128/mSystems.00583-21). Afterward, the taxonomic classification of 100,000 reads was performed using MegaBLAST (PMID: 26250111) with a manually curated and pre-processed database of genomes retrieved from the National Center for Biotechnology Information, following the METAnnotatorX2 manual [31,32].

### 2.6. Statistical Analysis

Descriptive statistics were expressed as number (%) of total, mean +/− SD or median (range).

Alpha-diversity was calculated using the Shannon–Wiener diversity index, providing information about taxa richness, taking into account the relative abundance (evenness).

Analyzing separately saliva and biopsy samples, beta-diversity analysis based on Bray–Curtis dissimilarity matrix was performed and graphically represented by principal coordinates analysis (PCoA) in 3D displaying cases and controls in different colors; Spearman correlation analysis (with FDR Benjamini&Hochberg correction) between cases and controls was performed to assess significant correlations between the bacterial species in the two groups; finally, sharing analysis between the single subjects were performed to test for a potential passage (transition) of bacterial species between the mouth and the stomach categorizing the samples into cases and controls. Independent *t*-tests with 1000 bootstraps were conducted using SPSS (version 2.0, IBM, www.ibm.com/software/it/analytics/spss, access 15 March 2026). Correlation analyses were performed in RStudio (RStudio Team, version 2.0), RStudio: Integrated Development for R. RStudio, PBC, Boston, MA, USA. www.rstudio.com) using the “Hmisc” and “corrplot” packages, generating Pearson correlation matrices. Post hoc power analyses were performed for alpha-diversity and bacterial abundance at an alpha-level of 0.05 (Medcalc^®^ Software Ltd., Ostend, Belgium, version 22.009).

## 3. Results

Of the 40 included subjects, 20 had a histological diagnosis of AIG and were defined as cases, and 20 had a normal gastric mucosa (*n* = 18) or antral-restricted, non-atrophic, non-active *H. pylori*-negative gastritis (with a normal corpus oxyntic mucosa, *n* = 2) and were defined as controls. The median age of cases and controls was 69 and 58 years, respectively, and females were 75% and 45%, respectively (*p* > 0.05). Also, the other features, such as nationality, region of residence, education level, employment status, lifestyle and oral hygiene items, were not different between groups (Table 1). Among AIG cases, corpus atrophy was severe, moderate, and mild in 55%, 35%, and 10%, respectively; intestinal metaplasia was present in 85%; the mean ± SD severity scores of the gastric corpus atrophy and intestinal metaplasia were 2.4 ± 0.8 and 1.2 ± 0.7, respectively, while the severity scores of chronic and active inflammatory infiltrates were 1.6 ± 0.7 and 0.1 ± 0.3, respectively. Supplementary Table S1 gives the detailed mean severity scores of histopathological changes in the gastric corpus and antral mucosa in cases and controls.

### 3.1. Alpha- and Beta-Diversity

Alpha-diversity, providing a descriptive measure of the diversity within the single samples, was analyzed by comparing the classified reads after filtering by Shannon–Wiener index, taking into consideration the abundance (evenness) and equity of the species distribution.

The bacterial abundance, expressed in classified reads after filtering, was significantly higher in saliva samples than in gastric biopsies in cases with AIG (median, IQR 159, 95–442 vs. 27,492, 12,842–39,663, *p* < 0.0001; mean ± SD 325.4 ± 305.3 vs. 27,167 ± 15,472, *p* < 0.0001) and in controls without AIG (median, IQR 113, 52–173 vs. 25,648, 12,252–42,751, *p* < 0.0001; mean ± SD 160.1 ± 149.7 vs. 30,974 ± 22,654, *p* < 0.0001).

Comparing cases and controls in the two body compartments, the bacterial abundance in the gastric biopsies was nearly two-fold higher in cases than in controls, albeit not reaching statistical significance (mean ± SD 325.4 ± 305.3 vs. 160.1 ± 149.7, *p* = 0.06, post hoc power = 0.47), while in saliva samples the abundance between cases and controls was similar (mean ± SD 27,167 ± 15,472 vs. 30,974 ± 22,654, *p* = 0.54, post hoc power = 0.09) (Supplementary Figure S1).

**Figure 1:**
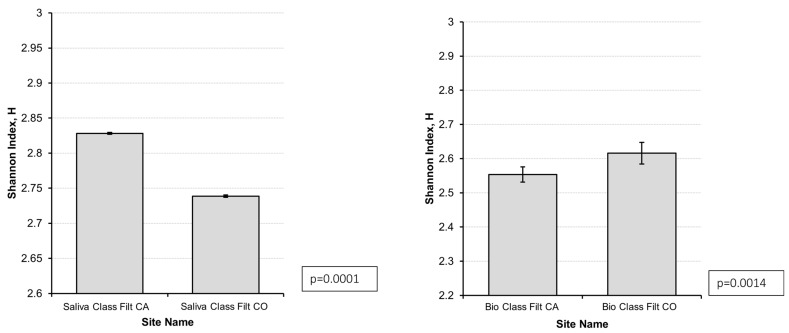
Biodiversity and relative abundance (evenness) as calculated by the Shannon–Wiener index in saliva samples (**left graph**) and gastric biopsies (**right graph**) of 20 cases (CA) with autoimmune gastritis and 20 controls (CO) without autoimmune gastritis.

To verify taxa richness by taking into account the relative abundance (evenness), the Shannon–Wiener diversity index was calculated and graphically represented in Figure 1. On gastric biopsy samples, AIG cases had a lower Shannon–Wiener diversity index than controls (2.55 vs. 2.61, *p* = 0.0014), showing a lower biodiversity and relative abundance (evenness) in cases, indicating dysbiosis. Conversely, on saliva samples, AIG cases showed a higher Shannon–Wiener diversity index than controls (2.83 vs. 2.73, *p* = 0.0000), indicating higher biodiversity and relative abundance (evenness) in cases than in controls.

**Figure 2:**
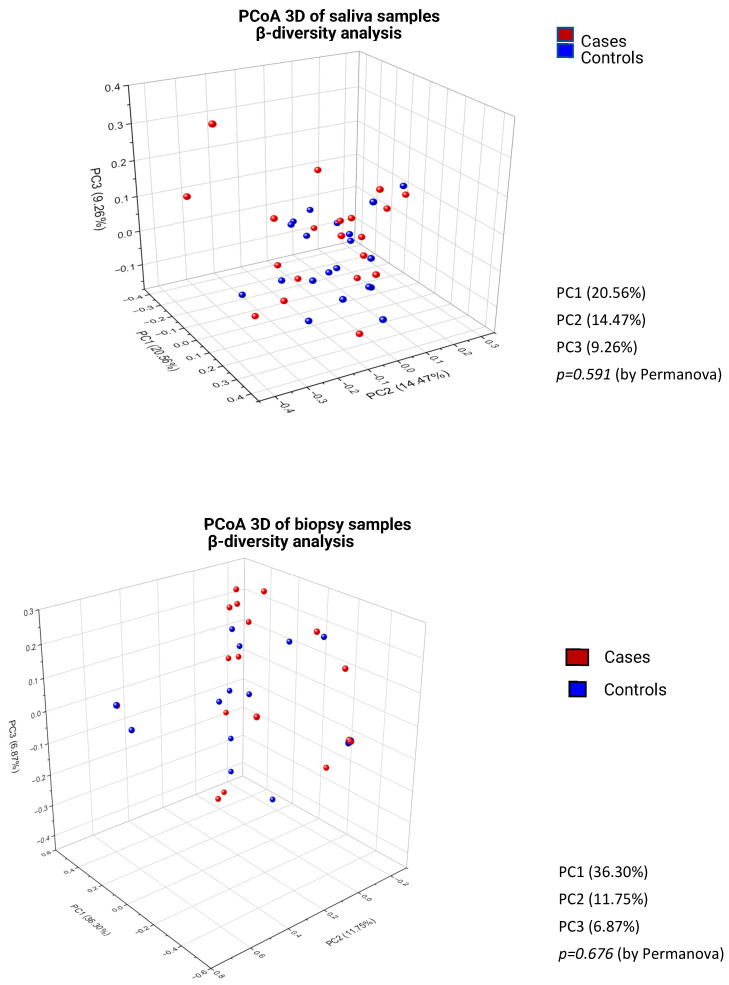
Beta-diversity analysis (based on Bray–Curtis dissimilarity matrix) of saliva (**upper graph**) and gastric biopsy (**lower graph**) of 20 cases with autoimmune gastritis (CA) and 20 controls without (CO) and represented by PCoA (principal coordinates analysis) in 3D. Cases are depicted in red and controls in blue. Beta-diversity between CA and CO was not statistically different, neither in saliva samples (*p* = 0.591) nor in gastric biopsies (*p* = 0.676), indicating that Bray–Curtis dissimilarity, mainly based on occurrence data (abundance), did not show significant differences between cases and controls in this specific study population.

Beta-diversity, representing the difference or distance between two ecosystems or sample groups, has been calculated by the Bray–Curtis dissimilarity matrix of the saliva and gastric biopsies of cases with AIG and controls and graphically displayed by PCoA in 3D. As shown in Figure 2, the beta-diversity between AIG cases and controls was not statistically different, neither in saliva samples (*p* = 0.591, post hoc power = 0.08) nor in gastric biopsies (*p* = 0.676, post hoc power = 0.06) indicating that Bray–Curtis dissimilarity, mainly based on occurrence data (abundance), did not show significant differences between cases and controls in this specific study population.

### 3.2. Taxonomy

In the whole study population, the total number of bacterial taxa with a prevalence of at least 2.5% retrieved in saliva samples was nearly four-fold higher than that in biopsy samples: *n* = 498 vs. *n* = 128 (ratio 3.9:1).

Overall, 17 bacterial taxa were retrieved in the saliva samples of all cases and controls (100% prevalence), such as *Streptococcus oralis*, *mitis*, *S. salivarius*, *S. parasanguinis*, *S. infantis*, *S. australis*, and *S. unknown species*, *Rothia mucilaginosa*, *R. dentocariosa*, and *R. unknown species*, *Prevotella jejuni* and *P. unknown species*, *Actinomyces unknown_species*, *Haemophilus parainfluenzae*, *Veillonella unknown_species*, *Granulicatella unknown_species* and *G. adiacens*, and other 12 bacterial taxa were highly prevalent (97.5%), amongst which *Prevotella histicola*, *P. melaninogenica*, *P. nigrescens*, *P. intermedia*, *and P. denticola*, *Streptococcus pseudopneumoniae*, *Gemella sanguinis*, *Haemophilus unknown species*, *Lancefieldella unknown species*, *Veillonella atypica*, *rogosae*, *V. dispar*, and *V. parvula* were common.

The top five bacterial taxa found in the stomach were *Cutibacterium acnes* (57.5%), *Streptococcus salivarius* (45%), *Streptococcus unknown species* (45%), *Streptococcus mitis* (40%), *Prevotella unknown species* (42.5%), while *Actinomyces unknown species*, *Prevotella melaninogenica*, and *Cutibacterium unknown species* had a lower prevalence of 37.5%, 37.5%, and 35%, respectively.

We then assessed for differences at the taxonomy level of bacterial species by performing a correlation analysis between variables by Spearman correlation analysis.

As shown in Table 2, in saliva samples, we found a significant positive association between AIG cases and *Gemella sanguinis* and *G. unknown_species*, *Chryseobacterium unknown species*, *Fusobacterium unknown species*, *F. nucleatum*, and *F. periodonticum*, and *Prevotella multisaccharivorax*, while in gastric biopsies, we found a significant positive association between AIG cases and *Streptococcus oralis*, *Gemella unknown species*, *Fusobacterium pseudoperiodonticum*, *Veillonella rogosae*, and *Gemella sanguinis*.

### 3.3. Sharing Analysis of Bacterial Taxa Between the ORAL and the Gastric Compartment

To test for a potential association of bacterial species between the mouth and the stomach, categorizing the samples into cases and controls, a sharing analysis of bacterial species between the oral and the gastric compartments of the single subjects was performed.

Supplementary Figure S2 shows in detail the shared bacterial taxa between saliva and gastric biopsies in cases with AIG and controls. As shown in Table 3, the most frequently shared bacterial taxa in cases with AIG were *Streptococcus oralis*, *Streptococcus mitis*, and *Prevotella histicola*. Notably, four bacterial taxa, namely *Fusobacterium pseudoperiodonticum*, *Gemella sanguinis*, *Gemella unknown species*, and *Veillonella rogosae*, were shared between the mouth and the stomach only in cases with AIG, but not in controls, likely favored by the non-acidic intragastric microenvironment in AIG.

Of the 20 cases with AIG, seven (35%) showed no sharing of bacteria between the mouth and the stomach, five (25%) shared only one taxon, while four (20%), two (10%), and two (10%) shared two, three and four taxa, respectively. Notably, *Gemella sanguinis* was never shared alone but always in association with other taxa, such as *Streptococci* or *Prevotella* (Table 4). Clinical (age, sex, clinical presentation, oral hygiene) or histological (severity of corpus atrophy, intestinal metaplasia) differences between AIG cases with or without sharing of taxa between the mouth and the stomach were not observed. 

## 4. Discussion

To our best knowledge, this is the first study that assessed at the same time the composition of the oral and gastric microbiota in patients with AIG, a well-known condition associated with impaired gastric acid secretion and hypochlorhydria, comparing them to subjects with normal gastric acid secretion.

From a quantitative point of view, the results of the current study showed that, in terms of classified reads after filtering, the oral microbiota was more abundant compared to the gastric microbiota, in cases with AIG and in controls as well (*p* < 0.0001). Also, the totally retrieved bacterial taxa in saliva samples were about four times higher than those in the gastric mucosa (*n* = 498 vs. 128, ratio 3.9:1). This may be interpreted as a consequence of the lower microbial colonization in the stomach than in the mouth and secondly due to the technical difficulty to extract and amplify bacterial DNA from gastric biopsies (mainly composed of human DNA), which may be extracted more easily from a liquid–viscous medium as the saliva.

The results of the current study further confirmed a lower Shannon–Wiener biodiversity index in cases with AIG than in controls. This shows a lower biodiversity and relative abundance (evenness), indicating gastric dysbiosis in AIG. This result keeps in step with previous reports [26,28]. The reduced bacterial complexity in AIG might be explained by the particular intragastric microenvironment in AIG characterized by chronic inflammation and the lack of the scavenging role of gastric acid due to the non-acidic intragastric pH, likely favoring the overgrowth of non-typical intragastric bacteria potentially swallowed with saliva and derived from the oral microbiota [17,27,28,33,34,35,36,37]. This concept has long been hypothesized but still awaits being proved by data. The current study, assessing at the same time the oral and gastric microbiota, is able to shed light on this hypothesis. Compared to controls, in the saliva of AIG cases, *Gemella sanguinis*, *Fusobacterium nucleatum*, *F. periodonticum* and *F. unknown species*, and *Prevotella multisaccharivorax* were significantly enriched, while in their gastric biopsies, *Streptococcus oralis*, *Gemella sanguinis*, *Fusobacterium pseudoperiodonticum*, and *Veillonella rogosae* were significantly enriched. Indeed, the most frequently shared bacterial taxa between the mouth and the stomach in AIG cases were *Streptococcus oralis*, *Streptococcus mitis*, and *Prevotella histicola*, and four bacterial taxa, namely *Fusobacterium pseudoperiodonticum*, *Gemella sanguinis* and *G. unknown species*, and *Veillonella rogosae* were shared between the mouth and the stomach exclusively in cases with AIG, but not in controls. These results firstly provide evidence that in AIG, the most frequently associated taxa were of oral origin. This is even more evident considering that the most frequently shared taxa between the mouth and the stomach in AIG were Streptococci e Prevotellae, and that the bacterial taxa exclusively shared between the mouth and the stomach in AIG were bacteria typically colonizing the oral cavity, such as *Fusobacterium pseudoperiodonticum*, *Gemella sanguinis*, and *Veillonella rogosae*. This phenomenon was not observed in controls with normal gastric acid secretion, thus strongly suggesting that the impaired gastric acid secretion and the consequently higher pH, probably together with the chronically inflamed gastric mucosa in AIG, might be determinant to permit the presence of these oral bacteria in the AIG stomach. Specifically, *Fusobacterium pseudoperiodonticum* is a recently described oral anaerobe originally isolated from the human tongue and subgingival plaque, supporting its oral origin [38]. It is well-documented for its role in multispecies biofilm organization. However, a recent study on *Fusobacterium nucleatum* reported marked resistance to highly acidic conditions (pH 1.5), attributed to membrane lipid composition—specifically the presence of erucic acid [39]. To date, there is no direct evidence demonstrating the same mechanism in *F. pseudoperiodonticum*. *Gemella sanguinis* is a recognized core oral commensal, and recent analyses indicate that it is present in the oral cavity, consistent with adaptation to oral microbial communities rather than to the gastric niche. In the stomach, *G. sanguinis* is present only under pathological conditions, such as a severe impairment of the gastric acid barrier [40]. *Veillonella rogosae* represents the taxon with the strongest functional rationale for oral–gastric translocation. It is an oral species frequently isolated from the dental plaque that participates in early biofilm formation [41]. Functionally, it utilizes lactate produced by other bacteria as an energy source and contributes to the reduction of nitrate to nitrite. These metabolic traits make it biologically plausible as a species that travels within oral bacterial consortia. Conversely, for these bacterial species, we could not identify direct experimental evidence demonstrating a defined mechanism of resistance to gastric acidity or stable survival in a normally acidic stomach. However, due to the cross-sectional study design, this result showing sharing taxa could reflect either translocation or independent colonization driven by similar selective pressures and can only prove the association of oral bacteria with the gastric microbiota, but not the causation of gastric dysbiosis.

The association of these oral bacteria in the context of AIG is interesting, as many of these bacteria have been described in the context of gastric cancer and its preneoplastic conditions. Pimentel-Nunes P et al. performed next-generation sequencing of full-length 16S rRNA gene profiling of gastric biopsy samples from patients with normal mucosa, advanced atrophic gastritis with intestinal metaplasia, and early gastric cancer [42] observing dysbiosis at all disease stages, but more significant at the intestinal metaplasia stage, with two bacterial genera progressively increasing from controls to intestinal metaplasia and cancer: Gemella (from 1.48% to 3.9%, *p* = 0.014) and Streptococcus (from 19.3% to 33.7%, *p* = 0.04) [39]. Gemella has further been found to be enriched in oral swab samples of patients with gastric cancer [43]. Streptococcus has been found to be enriched in the mouth and stomach of gastric cancer patients, as well as in gastric cancer tissues [37,44], and in *H. pylori*-negative gastric cancer patients [45]. Fusobacterium, a component of the oral cavity, has been associated with gastric cancer and precursor conditions independent of *H. pylori* infection [25,33,46,47,48]. Oral Fusobacterium was significantly enriched in cases with atrophic gastritis and intestinal metaplasia compared with controls, indicating oral dysbiosis in these subjects [17,49]. *Fusobacterium nucleatum* was found enriched in gastric cancer biopsy samples [49,50] and in the saliva of atrophic gastritis patients compared to healthy controls [51].

These data together with the findings of the current study strongly suggest that in hypochlorhydric precancerous conditions such as AIG, gastric dysbiosis is associated with the presence of bacteria originating from the mouth and that in the presence of impaired gastric acid secretion the composition of the oral microbiota may be relevant for the composition of the gastric microbiota focusing the attention to the oral hygiene and dental and periodontal health in these patients.

We are aware of some limitations of the current study. First, although we investigated a total number of 80 samples (40 saliva and 40 biopsy samples) of 20 AIG cases and 20 controls, sample numbers might still be too low for yielding statistical power, particularly in sub analyses; for this reason, post hoc power analyses were performed. Second, it is well known that diet may be a critical factor in microbiota studies; in our study, we did not apply a specific diet before the study, but none of the patients were on a special diet, and all patients were off antibiotics, pre- and probiotics and antisecretory drugs. Third, AIG is at increased risk of gastric cancer [6,52], but during the inclusion period, no new gastric cancers were diagnosed, and for that reason, AIG patients with gastric cancer could not be assessed in this study using freshly frozen saliva and biopsy samples for DNA extraction and subsequent microbiota analyses. Fourth, two controls had antral-restricted non-active non-atrophic *H. pylori* negative gastritis, but the exclusion of these two cases from a primary analysis did not change the results. Fifth, the cross-sectional design of the study does not allow for establishing whether oral dysbiosis precedes gastric dysbiosis or vice versa; to establish directionality, longitudinal studies or mechanistic experiments are needed.

One strength of the current study was that, unlike the traditional 16S rRNA sequencing, we here performed an untargeted, microbiome sequencing approach, allowing a broader bacterial and taxonomic profiling. The 16S rRNA gene as a phylogenetic marker is cost-effective and efficient, but it might be harmed by some biases, such as the hypervariable region choice or primer-dependent PCR amplification, leading to variance in the microbial composition assessment. Conversely, shotgun metagenomics is expensive but yields a broader taxonomic resolution and the possibility of detecting unknown species and strains of microbes at the cost of a higher bioinformatic burden [53].

In conclusion, the current study confirmed the presence of gastric dysbiosis in AIG. Oral taxa were more commonly associated with AIG and shared between the mouth and the stomach. These findings show in AIG that the oral microbiota is associated with gastric dysbiosis, focusing attention on the importance of oral eubiosis.

## Figures and Tables

**Table 1:** Study population: A total of 20 cases with autoimmune gastritis (AIG) and 20 cases without AIG (18 with normal gastric mucosa and 2 with antral-restricted non-atrophic non-active gastritis) as assessed by histopathology of gastric biopsies obtained during gastroscopy.

	Cases with AIG *n* = 20	Controls *n* = 20	*p*
Females, *n* (%)	15 (75)	9 (45)	0.0559
Age, years, median (range)	69.5 (26–83)	58 (25–84)	0.1196
**Nationality**, *n* (%)			
Italy	19 (95)	17 (85)	0.3480
Europe (not Italy)	0 (0)	2 (10)	
Out of Europe	1 (5)	1 (5)	
**Area of residence**, *n* (%)			
Central Italy	19 (95)	18 (90)	0.2201
Northern Italy	1 (5)	0 (0)	
Southern Italy	0 (0)	2 (10)	
**Level of education**, *n* (%)			
Lower Secondary (/Middle) School Diploma	9 (45)	7 (35)	0.5641
Upper Secondary (/High) School Diploma	7 (35)	6 (30)	
University Degree	4 (20)	7 (35)	
**Employment status**, *n* (%)			
Employed	16 (80)	16 (80)	0.7659
Unemployed	3 (15)	2 (10)	
Retired	1 (5)	2 (10)	
Alcohol consumption, *n* (%)	12 (60)	10 (50)	0.5302
Smoking habit (active or former)	8 (40)	10 (50)	0.3359
Previous PPI intake	0 (0)	7 (35)	0.0040
Previous anti-acid intake	2 (10)	11 (55)	0.0027
Previous FANS intake	7 (35)	9 (45)	0.5239
Family history of gastric cancer	5 (25)	2 (10)	0.2177
**Reason for undergoing gastroscopy**			
Reflux	0 (0)	7 (35)	<0.0001
Dyspepsia	0 (0)	7 (35)	
Anemia	0 (0)	6 (30)	
Surveillance of AAG	20 (100)	0 (0)	
Weekly physical activity <2 times/week	12 (60)	11 (55)	0.7521
Body mass index >25 kg/m^2^	6 (40)	6 (42.9)	0.8781
Candy consumption	13 (65)	8 (40)	0.1180
Previous pre/probiotic intake	2 (10)	5 (25)	0.2177
Dental prosthesis	12 (60)	8 (40)	0.2117
Recent dental treatments >12 months before	13 (65)	15 (75)	0.4956
Last professional dental hygiene session >12 months before	7 (35)	9 (45)	0.5239
Last dental visit >12 months before	10 (50)	13 (65)	0.3433
Mouthwash use	10 (50)	7 (35)	0.3434
Chewing gum consumption	2 (10)	6 (30)	0.1185
Previous periodontal diseases	6 (30)	5 (25)	0.7266

**Table 2:** Spearman correlation of saliva and gastric biopsy samples between cases (CA) with autoimmune gastritis (AIG) and controls without (CO) AIG.

**Biopsies**			
**Source**	**Taxa**	**Corr**	***p*-Value**
CA	*Streptococcus oralis*	0.3537	0.025
CA	*Gemella unknown_species*	0.3333	0.036
CA	*Fusobacterium pseudoperiodonticum*	0.3333	0.036
CA	*Veillonella rogosae*	0.3329	0.036
CA	*Gemella sanguinis*	0.3328	0.036
CO	*Streptococcus oralis*	−0.3537	0.025
CO	*Gemella unknown_species*	−0.3333	0.036
CO	*Fusobacterium pseudoperiodonticum*	−0.3333	0.036
CO	*Veillonella rogosae*	−0.3329	0.036
CO	*Gemella sanguinis*	−0.3328	0.036
**Saliva**			
**Source**	**Taxa**	**Corr**	***p-* Value**
CA	*Gemella sanguinis*	0.5674	0.000
CA	*Chryseobacterium unknown_species*	0.4628	0.003
CA	*Fusobacterium unknown_species*	0.4379	0.005
CA	*Prevotella multisaccharivorax*	0.4319	0.005
CA	*Fusobacterium nucleatum*	0.4202	0.007
CA	*Gemella unknown_species*	0.3640	0.021
CA	*Fusobacterium periodonticum*	0.3435	0.030
CA	*Brevibacterium unknown_species*	−0.3204	0.044
CA	*Microbacterium unknown_species*	−0.3303	0.037
CA	*Sharpea unknown_species*	−0.3327	0.036
CA	*Pseudobutyrivibrio unknown_species*	−0.3327	0.036
CA	*Moraxella unknown_species*	−0.3327	0.036
CA	*Bergeriella unknown_species*	−0.3327	0.036
CA	*Ottowia unknown_species*	−0.3327	0.036
CA	*Cryptobacterium unknown_species*	−0.3327	0.036
CA	*Bifidobacterium breve*	−0,3327	0,036
CA	*Petrimonas unknown_species*	−0.3327	0.036
CA	*Propionimicrobium unknown_species*	−0.3327	0.036
CA	*Mycolicibacterium unknown_species*	−0.3327	0.036
CA	*Anaerostipes unknown_species*	−0.3327	0.036
CA	*Lewinella unknown_species*	−0.3327	0.036
CA	*Sphingobacterium unknown_species*	−0.3327	0.036
CA	*Mycobacterium unknown_species*	−0.3327	0.036
CA	*Actinomyces gerencseriae*	−0.3426	0.030
CA	*Kocuria unknown_species*	−0.3627	0.021
CA	*Anaerobutyricum unknown_species*	−0.3769	0.017
CA	*Rhodococcus unknown_species*	−0.3769	0.017
CA	*Bifidobacterium longum*	−0.4061	0.009
CO	*Bifidobacterium longum*	0.4061	0.009
CO	*Anaerobutyricum unknown_species*	0.3769	0.017
CO	*Rhodococcus unknown_species*	0.3769	0.017
CO	*Kocuria unknown_species*	0.3627	0.021
CO	*Actinomyces gerencseriae*	0.3426	0.030
CO	*Sharpea unknown_species*	0.3327	0.036
CO	*Pseudobutyrivibrio unknown_species*	0.3327	0.036
CO	*Moraxella unknown_species*	0.3327	0.036
CO	*Bergeriella unknown_species*	0.3327	0.036
CO	*Ottowia unknown_species*	0.3327	0.036
CO	*Cryptobacterium unknown_species*	0.3327	0.036
CO	*Bifidobacterium breve*	0.3327	0.036
CO	*Petrimonas unknown_species*	0.3327	0.036
CO	*Propionimicrobium unknown_species*	0.3327	0.036
CO	*Mycolicibacterium unknown_species*	0.3327	0.036
CO	*Anaerostipes unknown_species*	0.3327	0.036
CO	*Lewinella unknown_species*	0.3327	0.036
CO	*Sphingobacterium unknown_species*	0.3327	0.036
CO	*Mycobacterium unknown_species*	0.3327	0.036
CO	*Microbacterium unknown_species*	0.3303	0.037
CO	*Brevibacterium unknown_species*	0.3204	0.044
CO	*Fusobacterium periodonticum*	−0.3435	0.030
CO	*Gemella unknown_species*	−0.3640	0.021
CO	*Fusobacterium nucleatum*	−0.4202	0.007
CO	*Prevotella multisaccharivorax*	−0.4319	0.005
CO	*Fusobacterium unknown_species*	−0.4379	0.005
CO	*Chryseobacterium unknown_species*	−0.4628	0.003
CO	*Gemella sanguinis*	−0.5674	0.000

**Table 3:** Top twelve shared bacterial taxa from saliva to gastric biopsy in cases (CA) with autoimmune gastritis and controls (CO).

Shared (From Saliva to Biopsy) Taxa Summary						
Group	CA		CO		CA vs. CO	
Taxonomy	Shared Count	Shared %	Shared Count	Shared %	(Shared % CA)—(Shared % CO)	(Shared % CA—Shared % CO)/(Shared % CA)
*Streptococcus oralis*	8	40%	2	10%	30%	75%
*Streptococcus mitis*	10	50%	6	30%	20%	40%
*Prevotella histicola*	5	25%	1	5%	20%	80%
*Fusobacterium pseudoperiodonticum*	4	20%	0	0%	20%	Only CA
*Gemella sanguinis*	4	20%	0	0%	20%	Only CA
*Gemella unknown_species*	4	20%	0	0%	20%	Only CA
*Veillonella rogosae*	4	20%	0	0%	20%	Only CA
*Streptococcus infantis*	4	20%	1	5%	15%	75%
*Streptococcus pseudopneumoniae*	4	20%	1	5%	15%	75%
*Porphyromonas unknown_species*	5	25%	2	10%	15%	60%
*Cutibacterium unknown_species*	4	20%	2	10%	10%	50%
*Prevotella nanceiensis*	4	20%	2	10%	10%	50%

**Table 4:** Sub analysis of the bacterial taxa shared between the oral and the gastric compartments only in the 20 cases with autoimmune gastritis.

Cases with Autoimmune Gastritis	20 (100%)
No sharing	7 (35%)
1 taxon shared (*n* = 2 SO, *n* = 1 PH, *n* = 2 SM)	5 (25%)
2 taxa shared (*n* = 2 SO + SM, *n* = 2 SM + PH)	4 (20%)
3 taxa shared (*n* = 2 GS + SO + SM)	2 (10%)
4 taxa shared (*n* = 2 GS + PH + SO + SM)	2 (10%

GS = *Gemella sanguinis* (mai sola); PH = *Prevotella histolitica*; SM = *Streptococcus mitis*; SO = *Streptococcus oralis*.

## Data Availability

Raw shallow shotgun sequencing data are available through the SRA under the study accession number PRJNA1404172. https://www.ncbi.nlm.nih.gov/bioproject/?term=PRJNA140172 (accessed on 26 January 2026).

## Supplementary Materials

The following supporting information can be downloaded at: https://www.mdpi.com/article/10.3390/microorganisms14040789/s1, Table S1: Mean of severity scores of histopathological changes of gastric corpus and antral mucosa in cases and controls. Figure S1: Comparison of classified reads (bacterial abundance) after % and n° filtering of gastric biopsy (*p* = 0.06) and saliva (*p* = 0.54) samples between cases and controls; CA = cases: n = 20 patients with autoimmune atrophic gastritis; CO = n = 20 controls without autoimmune atrophic gastritis. Figure S2: Shared bacterial taxa between saliva and gastric biopsies in cases with autoimmune atrophic gastritis and in controls. CA, cases; CO, controls.
