# Supplementary material for: Concomitant Assessment of Oral and Gastric Microbiota Composition in Autoimmune Gastritis Patients: A Case–Control Study
Source: Microorganisms. 2026 Mar 31;14(4):789. doi: 10.3390/microorganisms14040789 (PMC13118387; doi:10.3390/microorganisms14040789)
Supplement: Supplementary file 1 [file microorganisms-14-00789-s001.zip › microorganisms-4185865-supplementary figures.pdf]

Supplementary Figure S1. Comparison of classified reads (bacterial abundance) after % and n° filtering of gastric biopsy (p=0.06) and saliva (p=0.54) samples between cases and controls; CA = cases: n=20 patients with autoimmune atrophic gastritis; CO = n=20 controls without autoimmune atrophic gastritis. Data expressed as mean ± SD and analysed by Student's t-test

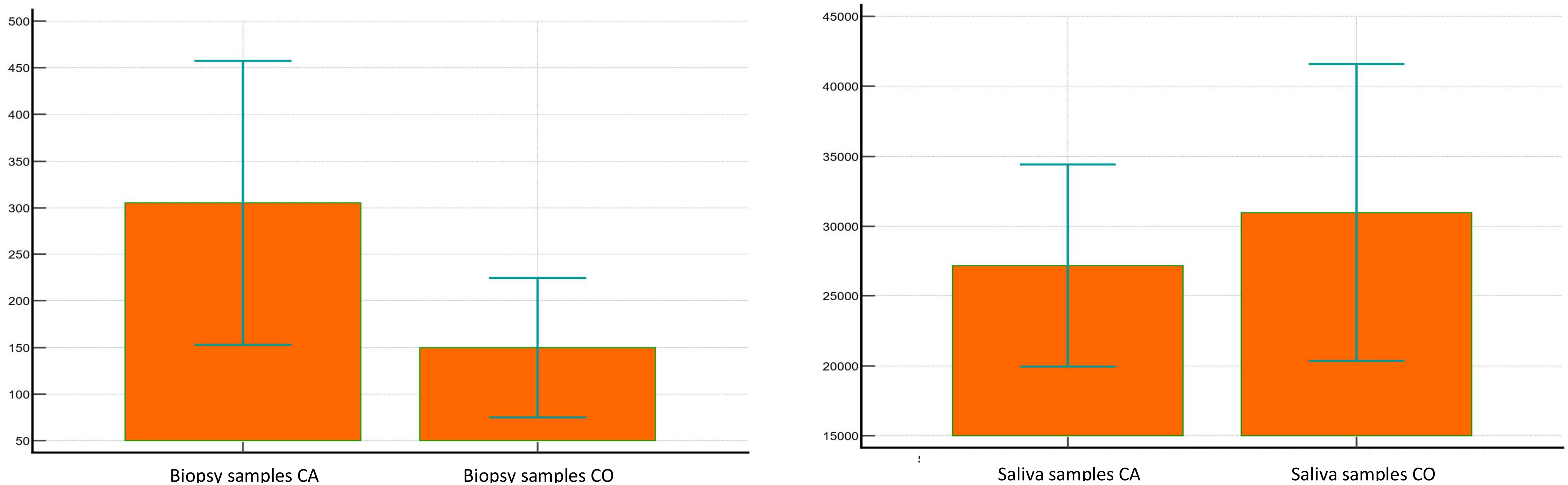

Figure S2. Shared bacterial taxa between saliva and gastric biopsies in cases with autoimmune atrophic gastritis and in controls. CA, cases; CO, controls.

Shared (from saliva to biopsy) taxa summary						
Group	CA		CO		CA vs. CO	
Taxonomy	Shared count	Shared %	Shared count	Shared %	(Shared % CA) - (Shared % CO)	(Shared % CA - Shared % CO)/(Shared % CA)
Streptococcus oralis	8	40,0%	2	10,0%	30,0%	75,00%
Streptococcus mitis	10	50,0%	6	30,0%	20,0%	40,00%
Prevotella histicola	5	25,0%	1	5,0%	20,0%	80,00%
Fusobacterium pseudoperiodonticum	4	20,0%	0	0,0%	20,0%	Only CA
Gemella sanguinis	4	20,0%	0	0,0%	20,0%	Only CA
Gemella unknown_species	4	20,0%	0	0,0%	20,0%	Only CA
Veillonella rogosae	4	20,0%	0	0,0%	20,0%	Only CA
Streptococcus infantis	4	20,0%	1	5,0%	15,0%	75,00%
Streptococcus pseudopneumoniae	4	20,0%	1	5,0%	15,0%	75,00%
Porphyromonas unknown_species	5	25,0%	2	10,0%	15,0%	60,00%
Cutibacterium unknown_species	4	20,0%	2	10,0%	10,0%	50,00%
Prevotella nanceiensis	4	20,0%	2	10,0%	10,0%	50,00%
Capnocytophaga leadbetteri	2	10,0%	0	0,0%	10,0%	Only CA
Rothia aeria	2	10,0%	0	0,0%	10,0%	Only CA
Veillonella infantium	2	10,0%	0	0,0%	10,0%	Only CA
Haemophilus haemolyticus	3	15,0%	1	5,0%	10,0%	66,67%
Veillonella unknown_species	3	15,0%	1	5,0%	10,0%	66,67%
Streptococcus unknown_species	10	50,0%	8	40,0%	10,0%	20,00%
Actinomyces unknown_species	8	40,0%	7	35,0%	5,0%	12,50%
Prevotella melaninogenica	8	40,0%	7	35,0%	5,0%	12,50%
Veillonella atypica	4	20,0%	3	15,0%	5,0%	25,00%
Capnocytophaga unknown_species	2	10,0%	1	5,0%	5,0%	50,00%
Granulicatella adiacens	2	10,0%	1	5,0%	5,0%	50,00%
Porphyromonas gingivalis	2	10,0%	1	5,0%	5,0%	50,00%
Prevotella veroralis	2	10,0%	1	5,0%	5,0%	50,00%
Streptococcus pneumoniae	2	10,0%	1	5,0%	5,0%	50,00%
Streptococcus timonensis	2	10,0%	1	5,0%	5,0%	50,00%
Bifidobacterium unknown_species	1	5,0%	0	0,0%	5,0%	Only CA
Enterococcus unknown_species	1	5,0%	0	0,0%	5,0%	Only CA
Eubacterium unknown_species	1	5,0%	0	0,0%	5,0%	Only CA
Granulicatella elegans	1	5,0%	0	0,0%	5,0%	Only CA
Haemophilus paraphrohaemolyticus	1	5,0%	0	0,0%	5,0%	Only CA
Mogibacterium diversum	1	5,0%	0	0,0%	5,0%	Only CA
Mogibacterium unknown_species	1	5,0%	0	0,0%	5,0%	Only CA
Neisseria elongata	1	5,0%	0	0,0%	5,0%	Only CA
Prevotella amnii	1	5,0%	0	0,0%	5,0%	Only CA
Prevotella denticola	1	5,0%	0	0,0%	5,0%	Only CA
Prevotella shahii	1	5,0%	0	0,0%	5,0%	Only CA
Streptococcus gordonii	1	5,0%	0	0,0%	5,0%	Only CA
Streptococcus halitosis	1	5,0%	0	0,0%	5,0%	Only CA
Tannerella forsythia	1	5,0%	0	0,0%	5,0%	Only CA
Veillonella parvula	1	5,0%	0	0,0%	5,0%	Only CA
Veillonella tobetsuensis	1	5,0%	0	0,0%	5,0%	Only CA
Streptococcus parasanguinis	5	25,0%	4	20,0%	5,0%	20,00%
Neisseria unknown_species	3	15,0%	2	10,0%	5,0%	33,33%
Streptococcus australis	3	15,0%	2	10,0%	5,0%	33,33%
Schaalia unknown_species	9	45,0%	9	45,0%	0,0%	0,00%
Streptococcus salivarius	9	45,0%	9	45,0%	0,0%	0,00%
Rothia mucilaginosa	5	25,0%	5	25,0%	0,0%	0,00%
Neisseria subflava	4	20,0%	4	20,0%	0,0%	0,00%
Haemophilus unknown_species	3	15,0%	3	15,0%	0,0%	0,00%
Haemophilus parahaemolyticus	2	10,0%	2	10,0%	0,0%	0,00%
Morococcus cerebrosus	2	10,0%	2	10,0%	0,0%	0,00%
Neisseria flavescens	2	10,0%	2	10,0%	0,0%	0,00%
Streptococcus rubneri	2	10,0%	2	10,0%	0,0%	0,00%
Aggregatibacter segnis	1	5,0%	1	5,0%	0,0%	0,00%
Gemella haemolysans	1	5,0%	1	5,0%	0,0%	0,00%
Lancefieldella unknown_species	1	5,0%	1	5,0%	0,0%	0,00%
Neisseria sicca	1	5,0%	1	5,0%	0,0%	0,00%
Prevotella nigrescens	1	5,0%	1	5,0%	0,0%	0,00%
Prevotella scopos	1	5,0%	1	5,0%	0,0%	0,00%
Solobacterium unknown_species	1	5,0%	1	5,0%	0,0%	0,00%
