# Supplementary material for: Concomitant Assessment of Oral and Gastric Microbiota Composition in Autoimmune Gastritis Patients: A Case–Control Study
Source: Microorganisms. 2026 Mar 31;14(4):789. doi: 10.3390/microorganisms14040789 (PMC13118387; doi:10.3390/microorganisms14040789)
Supplement: Supplementary file 1 [file microorganisms-14-00789-s001.zip › microorganisms-4185865-supplementary table.pdf]

Supplementary Table S1. Mean of severity scores of histopathological changes of gastric corpus and antral mucosa in cases and controls

Corpus mucosa	Cases			Controls			
	n	Mean	SD	n	Mean	SD	*p* [a]
Atrophy	20	2,4000	0,8208	20	0,0000	0,0000	<0,0001
Activity	20	0,1000	0,3078	20	0,0000	0,0000	0,8401
Chronic inflammation	20	1,6000	0,6806	20	0,2500	0,5501	<0,0001
Intestinal metaplasia	20	1,2500	0,7164	20	0,0000	0,0000	<0,0001

[a] Student's t-test

Antral mucosa	Cases			Controls			
	n	Mean	SD	n	Mean	SD	*p* [a]
Atrophy	20	0,0000	0,0000	20	0,0000	0,0000	0,1023
Activity	20	0,0000	0,0000	20	0,0000	0,0000	1,0000
Chronic inflammation	20	0,0000	0,0000	20	0,1000	0,2469	0,9010
Intestinal metaplasia	20	0,0000	0,0000	20	0,0000	0,0000	1,0000

[a] Student's-test
